# Haematological changes in rabbits (*Oryctolagus cuniculus* f. *domesticus*) in the course of pregnancy

**DOI:** 10.1590/1984-3143-AR2021-0013

**Published:** 2021-07-12

**Authors:** Maria Chmurska-Gąsowska, Bartosz Bojarski, Leszek Szała

**Affiliations:** 1 Institute of Veterinary Sciences, University Center of Veterinary Medicine JU-UA, University of Agriculture in Krakow, Krakow, Poland; 2 Institute of Ichthyobiology and Aquaculture in Gołysz, Polish Academy of Sciences, Chybie, Poland; 3 Department of Mathematics, Faculty of Chemical Engineering, University of Chemistry and Technology Prague, Prague, Czech Republic

**Keywords:** rabbits, physiology, haematology, erythrogram, pregnancy

## Abstract

The analysis of haematological parameters is an important element of the assessment of the physiological condition of animals. Haematological parameters may change both under the influence of various external factors, and in the course of normal pregnancy, which has been found in various species of mammals, including rabbits. Our study showed statistically significant (p<0.05) changes in basic haematological parameters: RBC (decrease; 5.87±0.48 at day 15 vs. 5.42±0.32 T/L at day 26), MCH (increase; 1.35±0.04 before matching vs. 1.41±0.03 fmol at day 26), RDW (decrease; 15.77±1.80 at day 15 vs. 14.27±1.57% at day 26) MPV (increase; 5.17±0.31 at day 15 vs. 5.92±0.70 fL at day 26), WBC (decrease; 8.60±2.57 at day 15 vs. 4.94±0.88 G/L at day 26) and PLT (decrease; 398.17±91.67 before matching vs. 271.67±61.72 G/L at day 26) in Termond White rabbits and RBC (decrease; 6.18±0.68 before matching vs. 5.68±0.54 T/L at day 26), Hb (decrease; 8.00±0.90 before matching vs. 7.32±0.71 mmol/L at day 26), MCH (decrease; 1.32±0.05 at day 15 vs. 1.29±0.04 fmol at day 26) and WBC (decrease; 9.62±1.81 before matching vs. 5.85±2.23 G/L at day 26 as well as 9.58±2.35 at day 15 vs. 5.85±2.23 G/L at day 26) in Popielno White rabbits. Moreover, in the Popielno White rabbits we recorded a significant (p<0.05) decrease in the percentage of irregular erythrocytes at the end of pregnancy (11.00±10.02 at day 15 vs. 3.00±4.94 at day 26). The changes appear to be physiological but should be considered in studies using rabbits as model organisms.

## Introduction

In many species, blood volume increases during pregnancy (Nuwayhid, 1979; Hart et al., 1985; Barron, 1987; Soma-Pillay et al., 2016). In women, from as early as the sixth week of pregnancy, the volume of circulating blood increases slowly to reach 150% of its baseline at a more advanced stage (Bernstein et al., 2001; Horowitz et al., 2013). The phenomenon of increased blood volume leads to ‘physiological pregnancy anaemia’ which is due to the fact that plasma volume increases more quickly than red cell mass (Horowitz et al., 2013). Anaemia in the course pregnancy has been found in pregnant dogs (Bussabarger et al., 1938) and ewes (Mostello et al., 1991). A study conducted by Nuwayhid (1979) showed a 62% increase in plasma volume in the third trimester in rabbits. Mizoguchi et al. (2010) on pregnant rabbits showed that from the 18^th^ day of pregnancy onwards, there is a decrease in the total red blood cells count (RBC), haemoglobin (Hb) concentration and the haematocrit (Ht) value. The mean corpuscular volume (MCV), mean corpuscular haemoglobin (MCH) and the mean corpuscular haemoglobin concentration (MCHC) did not change (Mizoguchi et al., 2010). However, Wells et al. (1999) observed an increase in MCV in advanced pregnancy in rabbits of the same breed. Studies carried out by Mizoguchi et al. (2010) showed an increase in the reticulocyte count in female rabbits on the 13^th^-18^th^ day of gestation. This can probably be explained by the increased demand for erythrocytes due to placental development and foetal organogenesis, as found in the case of rats (De Rijk et al., 2002). In rabbits, the white blood cell (WBC) count increases at the beginning of pregnancy (Kim et al., 2002) to start decreasing significantly after 24 days, which is probably associated with an increase in blood volume (Kim et al., 2002; Mizoguchi et al., 2010). The increase in WBC count up to the 16th day of pregnancy in rabbits was shown by Wells et al. (1999). Similarly, an increase in WBC at the beginning of pregnancy was also observed in rats (Papworth and Clubb, 1995). Dogs and cats also exhibit an increase in WBC, but this is more consistent and continues until late pregnancy (Dimco et al., 2013; Simsek et al., 2015). During pregnancy in women, the number of leukocytes increases with the development of pregnancy and the resulting leukocytosis is explained by physiological stress. The highest value of WBC has been observed in the second trimester, but this is a result of a high percentage of neutrophils coupled with a low percentage of lymphocytes (Miresan et al., 2017). Studies conducted by Kim et al. (2002) observed that after the initial slight increase up to the fourth day of pregnancy, the number of lymphocytes began to decrease, reaching a nadir in the 24^th^ day of pregnancy. The neutrophil count fluctuated up to the 8^th^ day of pregnancy, decreased thereafter, similarly to lymphocytes, and reached a nadir on the 24^th^ day of pregnancy. No eosinophils (present before) were detected on the 24^th^ day of pregnancy. In a study conducted by Mizoguchi et al. (2010), the number of platelets (PLT) in pregnant rabbits increased between the 18^th^ and 25^th^ day of pregnancy. In pregnant women, a decrease in PLT count was observed over the course of pregnancy, with an increasing tendency to aggregate, especially in the last eight weeks (Fay et al., 1983). The interpretation of erythrogram, i.e. analysis of red blood cell morphology based on blood smear evaluation, plays a key role in detailed haematological examination (Witeska et al., 2011; Bojarski et al., 2018; Burgos-Aceves et al., 2019; Sula et al. 2020). Changes in erythrogram occur in different species of mammals, including rabbits, in different pathological states (Christopher et al., 2014; Kritsepi-Konstantinou and Oikonomidis, 2016). It has been demonstrated that erythrocyte deformability is impaired during an acute inflammatory response (Silva-Herdade et al., 2016). Moreover, erythrocytes of mammals can be used to assess xenobiotic toxicity (e.g. Pagano and Faggio, 2015). It has been established that during pregnancy red blood cells in rabbits undergo metabolic and biochemical changes (Ozegbe, 2001). It is not known, however, whether these changes translate into the occurrence of abnormally shaped erythrocytes. It seems important to investigate this issue to determine whether any changes observed in erythrogram (erythrocyte morphology) in pregnant rabbits are a result of a disease process or haematological changes occurring during normal pregnancy. Given the above, the purpose of this work was to: 1) add to the literature concerning changes in basic haematological parameters in rabbits during normal pregnancy 2) investigate whether pregnancy influences erythrogram (erythrocyte morphology based on a blood smear) 3) determine whether haematological changes (including any changes in erythrogram) depend on the breed of the rabbit.

## Materials and Methods

The experiment was carried out in the summer months, on the premises of the Experimental Station of the Department of Genetics and Methods of Animal Improvement of the University of Agriculture in Krakow, in the district of Przegorzały. Before conducting the study, we obtained permission from the 2^nd^ Local Ethics Committee (Resolution No. 146/2018 of 12.04.2018). The rabbits used for the experiment were 12 healthy female domestic rabbits (*Oryctolagus cuniculus* f. *domesticus*) aged 15-24 months, representing 2 breeds: the Termond White (6 specimens) and the Popielno White (also 6 specimens). The animals belonged to the breeding herd of the aforementioned breeder, registered at the National Animal Breeding Centre under the following numbers: K017 for the Popielno White rabbit and K026 for the Termond White rabbit.

The rabbits were kept individually, in wooden cages (80 cm x 70 cm x 65 cm) with bedding, whose dimensions met the current standards. The cages stood in an insulated hall (12 m x 25 m) equipped with a water system (nipple drinkers) and forced ventilation. The animals were exposed to 14L:10D (14 h light and 10 h darkness), with light intensity of 60 lux. The average temperature on the farm was 15 °C-18 °C. The humidity was kept at 55%. The animals had permanent access to water and complete granulated feed and were under constant veterinary care. The cages and feeders were systematically cleaned and disinfected according to the animals' needs. The rabbits were vaccinated against rabbit haemorrhagic disease RHD1 and myxomatosis according to the vaccination schedule.

The blood for haematological analysis was taken three times (3 days before mating, and on the 15^th^ and 26^th^ day of pregnancy – sampling I, II and III) from the saphenous vein, using 0.8 x 40 mm needle, to PP tubes with EDTA, V – 1ml (morphology). Next, smears were prepared on microscope slides, left to dry and stained with Diff Quick stains according to the instructions provided by the manufacturer.

**Figure 1:**
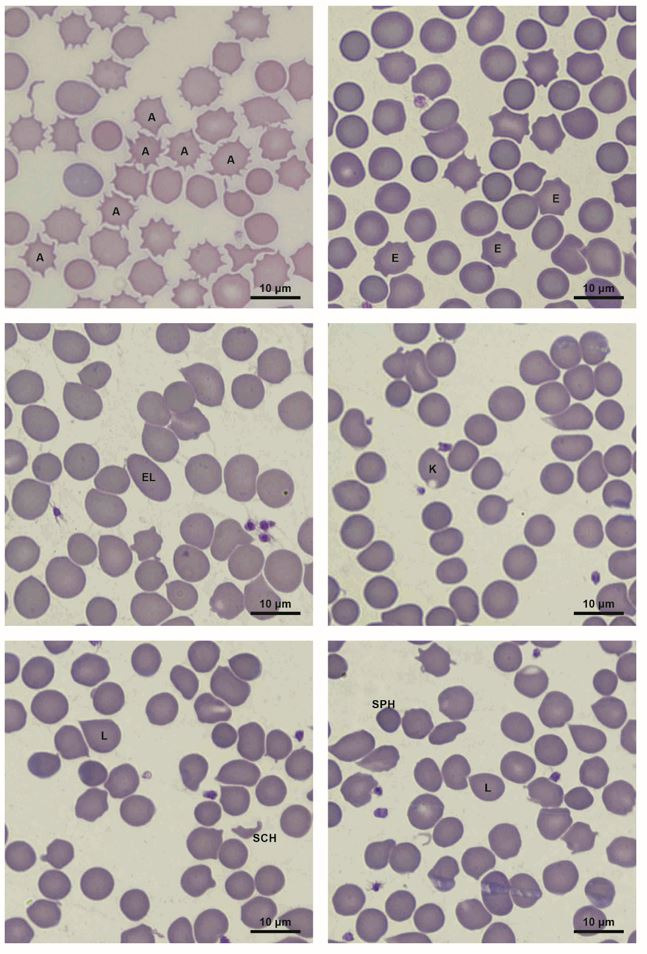
Erythrocyte morphology changes in rabbits (A – acanthocyte, E – echinocyte, EL – eliptocyte, K – keratocyte, L – lacrimocyte, SCH – schistocyte, SPH – spherocyte).

Haematological examination was performed with Mindray BC-5300Vet analyser employing flow cytometry, with a semiconductor laser using Comray reagents. The samples were tested for the following parameters: RBC count, Hb concentration, Ht value, MCV, MCH, MCHC, RDW (red cell distribution width), MPV (mean platelet volume), WBC count, percentage composition of individual leukocyte types (leukogram), and PLT count. The smears were used to analyse erythrocyte morphology (Figure 1). In order to determine the erythrogram, 300 erythrocytes were analysed each time. The erythrograms were determined manually using Nikon Eclipse Ci light microscope (1000 x magnification).

Since the distribution of the results did not match the normal distribution, the results were analysed using the Friedman test along with a post hoc test using Statistica 13.3. The level of significance was set at α = 0.05.

## Results

In Termond White rabbits, the RBC count was statistically significantly higher on the 15^th^ day of pregnancy in comparison to the 26^th^ day of pregnancy. A similar phenomenon was observed for WBC. In the case of MCH, a statistically significant difference was observed between the pre-pregnancy blood sampling and day 26 sampling (significantly higher value at the end of pregnancy). The RDW value was significantly lower in sampling III compared to the value recorded in sampling II. The MPV value was significantly higher on the 26^th^ day of pregnancy in comparison to the 15^th^ day. The PLT count on the 26^th^ day was significantly decreased in comparison to the sampling performed before mating (Table 1). A statistically significant decrease in RBC and Hb values was found in Popielno White rabbits on the 26^th^ day of pregnancy in comparison to the pre-mating blood sample. There was also a significant decrease in MCH in sampling III compared to sampling II, as well as a significant decrease in WBC in sampling III in comparison to both the sampling performed before mating and the blood collection carried out on the 15^th^ day of pregnancy (Table 2).

**Table 1:** Basic haematological parameters in Termond White rabbits.

**Parameter**	**Sampling I**	**Sampling I**	**Sampling III**
	**(mean±SD)**	**(mean±SD)**	**(mean±SD)**
RBC (T/L)	5.57±0.39^AB^	5.87±0.48^A^	5.42±0.32^B^
Hb (mmol/L)	7.50±0.38	8.13±0.64	7.63±0.50
Ht (L/L)	0.39±0.03	0.42±0.04	0.39±0.02
MCV (fL)	69.18±3.83	71.15±1.89	72.27±1.45
MCH (fmol)	1.35±0.04^A^	1.39±0.05^AB^	1.41±0.03^B^
MCHC (mmol/L)	19.63±1.22	19.53±0.58	19.52±0.18
RDW (%)	14.22±2.16^AB^	15.77±1.80^A^	14.27±1.57^B^
MPV (fL)	5.52±0.31^AB^	5.17±0.31^A^	5.92±0.70^B^
WBC (G/L)	7.18±2.28^AB^	8.60±2.57^A^	4.94±0.88^B^
Lymphocytes (%)	63.00±10.00	61.67±12.34	55.17±13.54
Neutrophils (%)	29.17±11.07	27.67±13.34	35.83±12.69
Monocytes (%)	4.17±2.40	4.67±3.33	3.17±0.98
Eosinophils (%)	0.67±1.03	2.50±3.08	1.50±0.84
Basophils (%)	3.00±2.45	3.50±2.51	4.33±2.66
PLT (G/L)	398.17±91.67^A^	345.33±161.28^AB^	271.67±61.72^B^

Values in rows marked with different letters (A or B) differ significantly (p<0.05).

**Table 2:** Basic haematological parameters in Popielno White rabbits.

**Parameter**	**Sampling**	**Sampling I**	**Sampling III**
	**(mean±SD)**	**(mean±SD)**	**(mean±SD)**
RBC (T/L)	6.18±0.68^A^	6.00±0.69^AB^	5.68±0.54^B^
Hb (mmol/L)	8.00±0.90^A^	7.92±0.80^AB^	7.32±0.71^B^
Ht (L/L)	0.41±0.04	0.39±0.04	0.37±0.04
MCV (fL)	65.92±1.64	65.13±1.58	65.10±2.33
MCH (fmol)	1.30±0.04^AB^	1.32±0.05^A^	1.29±0.04^B^
MCHC (mmol/L)	19.72±0.55	20.27±0.48	19.83±0.25
RDW (%)	13.15±0.66	13.45±0.77	13.22±1.05
MPV (fL)	5.63±0.74	5.18±0.45	5.68±0.18
WBC (G/L)	9.62±1.81^A^	9.58±2.35^A^	5.85±2.23^B^
Lymphocytes (%)	69.67±14.11	67.17±3.71	61.67±9.91
Neutrophils (%)	24.33±11.94	24.17±5.67	32.00±9.96
Monocytes (%)	2.17±2.04	3.00±1.67	3.33±2.73
Eosinophils (%)	1.50±1.52	1.17±1.33	0.33±0.82
Basophils (%)	2.33±2.34	4.50± 4.28	2.67±2.42
PLT (G/L)	338.67±144.95	371.33±108.47	269.50±98.90

Values in rows marked with different letters (A or B) differ significantly (p<0.05).

The differences between the erythrograms of the two breeds were not statistically significant, except for the number of irregular erythrocytes (also called ‘poikilocytes’) in the case of the Popielno White rabbits, a significant decrease in the percentage of these cells was noted on the 26^th^ day of pregnancy compared to the 15^th^ day of pregnancy (Table 3 and 4).

**Table 3:** Erythrogram in Termond White rabbits.

**Type of erythrocytes**	**Sampling I**	**Sampling II**	**Sampling III**
	**(mean±SD)**	**(mean±SD)**	**(mean±SD)**
unchanged erythrocytes	269.67±18.79	227.17±49.73	265.17±24.94
acanthocytes	4.83±4.88	23.00±33.03	5.83±10.46
echinocytes	7.17±8.11	13.17±14.36	13.67±20.20
eliptocytes	0.17±0.41	2.00±1.79	1.00±2.45
keratocytes	0.17±0.41	0.00±0.00	0.00±0.00
lacrimocytes	6.17±5.60	7.50±9.27	1.67±2.07
schistocytes	0.17±0.41	0.17±0.41	0.33±0.82
spherocytes	4.17±4.40	7.50±15.51	6.00±5.10
irregular erythrocytes	7.50±9.40	19.50±18.13	6.33±4.80

**Table 4:** Erythrogram in Popielno White rabbits.

**Type of erythrocytes**	**Sampling I**	**Sampling II**	**Sampling III**
	**(mean±SD)**	**(mean±SD)**	**(mean±SD)**
unchanged erythrocytes	285.33±9.97	271.00±21.31	279.33±16.75
acanthocytes	2.67±3.92	5.33±3.67	9.67±8.16
echinocytes	3.50±2.95	6.33±4.84	5.33±3.67
eliptocytes	0.17±0.41	0.50±0.84	0.00±0.00
keratocytes	0.00±0.00	0.00±0.00	0.00±0.00
lacrimocytes	2.17±3.06	4.17±3.97	0.67±1.21
schistocytes	0.17±0.41	0.17±0.41	0.50±0.84
spherocytes	1.67±1.97	1.50±2.35	1.50±1.97
irregular erythrocytes	4.33±5.35^AB^	11.00±10.02^A^	3.00±4.94^B^

Values in rows marked with different letters (A or B) differ significantly (p<0.05).

## Discussion

Our study revealed quite numerous changes in basic haematological parameters. The changes were detected in: RBC (decrease), Hb (decrease), MCH (decrease or increase), MPV (increase), WBC (decrease), and PLT (decrease). Also, Kim et al. (2002) revealed that in New Zealand White rabbits the basic haematological parameters change during pregnancy. RBC counts and Hb concentrations after 20-28 gestational days were lower than those of non- pregnant rabbits. These values fluctuated slightly between 0 and 12 days and subsequently decreased to reach a nadir on either the 24^th^ or 28^th^ day. Mizoguchi et al. (2010) revealed that in rabbits of the same breed, RBC count, Hb concentration and Ht value began to decrease during organogenesis and continued to decrease thereafter. Moreover, significantly lower RBC values in pregnant Angora rabbits as compared to non-pregnant individuals were noted by Cetin et al. (2009). A similar phenomenon was observed by Sukar et al. (2020) and AL-Eissa (2011). The reticulocyte count significantly increased during organogenesis and decreased thereafter. Haneda et al. (2010) observed that in pregnant Japanese White rabbits, RBC count, Ht value and Hb concentration were significantly lower on the 28^th^ day of pregnancy than in non-pregnant rabbits. Similarly, in our study, RBC was significantly reduced at the end of pregnancy in both breeds. Significant reduction of haemoglobin concentration was also observed, but only in Popielno White rabbits. Kim et al. (2002) observed that MCV in pregnant rabbits increased gradually during the course of gestation and was greater on the 24^th^ gestational day than in non-pregnant rabbits. No similar relationship was noticed in our own research. In the study performed by Kim et al. (2002), WBC and lymphocyte counts in pregnant New Zealand White rabbits on the 24^th^ day of pregnancy were lower than those of non-pregnant individuals. Similarly, in our study, the WBC count in Popielno White rabbits measured on the 26^th^ day of pregnancy was significantly lower than the WBC value detected in the sampling performed before mating as well as lower than the WBC count detected in the sampling performed on the 15^th^ day of pregnancy. This also applied to Termond White rabbits, whose WBC values were significantly lower towards the end of pregnancy, albeit only in comparison to sampling II. A significant decrease in WBC in pregnant rabbits as compared to non-pregnant ones was also reported by Cetin et al. (2009) and Al-Eissa (2011). Mizoguchi et al. (2010) revealed that in New Zealand White rabbits, white blood cell parameters (except for neutrophils) showed significant decreases during the foetal growth stage. In a study performed by Haneda et al. (2010), pregnant Japanese White rabbits showed lower WBC values, eosinophil and basophil counts in comparison to non-pregnant animals. In our study, we did not observe significant changes in eosinophil and basophil percentages. The statistically significant reduction in RBC and WBC values observed in our research may be a result of increased blood volume during pregnancy (Nuwayhid, 1979; Hart et al., 1985; Barron, 1987, Soma-Pillay et al. 2016). In the study conducted by Kim et al. (2002), the PLT counts on 24-28^th^ day were significantly lower than those of non-pregnant individuals. Similarly, in our study, the PLT count in Termond White rabbits was significantly reduced on the 26^th^ day of pregnancy in comparison to the pre-mating sampling.

Basic haematological parameters may change during normal pregnancy not only in rabbits, but also in other species of mammals. Waziri et al. (2010) studied the effects of pregnancy on haematological indices in Sahel goats and showed that the mean values of RBC and WBC increased significantly in the 16^th^ and 20^th^ weeks of gestation (third trimester), while other parameters were unchanged. Honda et al. (2008) analysed haematological changes in rats during pregnancy and revealed that RBC, Hb and Ht values decreased on days 7, 14, 17 and 21, while MCH, MCHC and neutrophil counts and rate increased on days 14, 17 and 21 as compared with non-pregnant rats. Poljicak-Milas et al. (2009a) tested the haematological parameters in the blood of pregnant and non-pregnant red deer (*Cervus elaphus*) and fallow deer (*Dama dama*). A marked reduction of RBC, Hb and Ht values was detected in pregnant animals of both of these species. Pregnant red deer hinds had a lower lymphocyte count than non-pregnant ones, while pregnant fallow deer hinds had more than twice the lymphocyte count in their blood as non-pregnant animals. Roy et al. (2010) studied the haematological profile of Sahiwal cows during gestation period. The RBC value and Hb content decreased, while the total WBC and the percentage of segmented neutrophils increased after the second gestation period. Bonelli et al. (2016) observed that in jennies (*Equus asinus*) RBC and Ht values were higher in late pregnancy than at foaling and during lactation. The WBC count was higher at foaling than during late pregnancy and lactation. According to the authors, this could be related to the release of cortisol and catecholamine during delivery. The PLT trend showed lower values from delivery to the first 2 months of lactation compared to late gestation (Bonelli et al., 2016).

In the case of animals, erythrogram analysis is of great diagnostic importance (Witeska et al., 2011; Bojarski et al., 2018). The available literature data concerns the morphometry of erythrocytes in New Zealand white rabbits (Poljicak-Milas et al., 2009b) and the occurrence of poikilocytosis accompanying various organ and systemic diseases in rabbits (Christopher et al., 2014). Christopher et al. (2014) analysed blood smears from 482 rabbits and found ‘fragmented red blood cells’ (schistocytes, microcytes, keratocytes, spherocytes; >0.5% of RBC) in 6% of diseased and 0% of healthy rabbits. The authors revealed that erythrocyte fragmentations were more severe in individuals suffering from inflammatory diseases and malignant neoplasia in comparison to healthy individuals.

Our study revealed that apart from normal erythrocytes, the rabbits’ blood also contained acanthocytes, echinocytes, elliptocytes, keratocytes, lacrimocytes, schistocytes, spherocytes and irregular red blood cells, which could not be classified into any of the above-mentioned categories (‘poikilocytes’). As all of these types of blood cells were present in both pre-mating and pregnant rabbits, and the animals included in the study were healthy, the presence of altered erythrocytes seems to be physiological. To our knowledge, there is no literature data concerning possible changes in the morphology of red blood cells during pregnancy in mammals. Our study did not reveal any clear influence of pregnancy on erythrocyte shape, except for a decrease in the percentage of irregular blood cells in the Popielno White rabbits.

## Conclusion

Our study confirms earlier literary accounts of changes to basic hematological parameters observed in rabbits during pregnancy, with more of the recorded index values being decreased rather than increased. The decrease in red and white blood cell count is probably linked to increased blood volume. The observed changes were dependent on the animal's breed, although they partly overlapped in both breeds. The changes appear to constitute a physiological response, which should be taken into account in experimental studies using rabbits as a model organism to correctly interpret the obtained results.
